# Identification of vulnerable carotid plaque with histologically validated CT-derived plaque maps

**DOI:** 10.1259/bjr.20220982

**Published:** 2023-05-15

**Authors:** Daniel Rhys Obaid, Ike Okonji, Suk F Cheng, Argyrios A Giannopoulos, Pragash Kamalathevan, Julian Halcox, Manuel Rodriguez-Justo, Toby Richards

**Affiliations:** 1 Swansea University Medical School, Swansea, UK; 2 University College London Hospitals NHS Foundation Trust, London, UK; 3 University of Oxford, Oxford, UK; 4 Department of Surgery, University of Western Australia, Perth, Australia

## Abstract

**Objectives:**

Ruptured carotid plaque causes stroke, but differentiating rupture-prone necrotic core from fibrous tissue with CT is limited by overlap of X-ray attenuation. We investigated the ability of CT-derived plaque maps created from ratios of plaque/contrast attenuation to identify histologically proven vulnerable plaques.

**Methods:**

Seventy patients underwent carotid CT angiography and carotid endarterectomy. A derivation cohort of 20 patients had CT images matched with histology and carotid plaque components attenuation defined. In a validation cohort of 50 patients, CT-derived plaque maps were compared in 43 symptomatic *vs* 40 asymptomatic carotid plaques and accuracy detecting vulnerable plaques calculated.

**Results:**

In 250 plaque areas co-registered with histology, the median attenuation (HU) of necrotic core 43(26-63), fibrous plaque 127(110-162) and calcified plaque 964 (816–1207) created significantly different ratios of plaque/contrast attenuation. CT-derived plaque maps revealed symptomatic plaques had larger necrotic core than asymptomatic (13.5%(5.9–33.3) *vs* 7.4%(2.3–14.3), *p* = 0.004) with large necrotic core predicting symptoms (area under ROC curve 0.68, *p* = 0.004). Twenty-four of 47 carotid plaques were histologically classified as most vulnerable (Starry-Type VI). Plaque maps revealed Type VI plaques had a greater necrotic core volume than Type IV/V plaques and a necrotic core/fibrous plaque ratio >0.5 distinguished Type VI plaques with sensitivity 75.0% (55.1–88.0) and specificity of 39.1% (22.2–59.2).

**Conclusions:**

Carotid plaque components can be differentiated by CT using a ratio of plaque/contrast attenuation. CT-derived plaque map volumes of necrotic core help distinguished the most vulnerable plaques.

**Advances in knowledge:**

CT-derived plaque maps based on plaque/contrast attenuation may provide new markers of carotid plaque vulnerability.

## Introduction

Carotid atherosclerosis is a common and modifiable cause of ischaemic stroke and intervention by carotid endarterectomy or stenting can reduce patient risk of future stroke or death.^1^ Current guidelines recommend intervention in recently symptomatic patients and support enrollment of lower risk (asymptomatic) patients into clinical trials.^2,3^ The risk of carotid atherosclerosis to cause stroke has historically focused on patient’s symptoms and degree of carotid luminal stenosis. However, the mechanism of carotid plaque instability and rupture is associated with a plaque morphology of large lipid cores with thin, inflamed rupture-prone caps.^4^ Assessment of the carotid plaque to identify those at high-risk features has been performed with ultrasound, PET, MRI and CT.^5,6^ However, utilization of routinely available imaging is preferable for widespread utilization. Carotid CT angiography is routine practice to investigate patients with carotid artery stenosis and offers a potentially promising avenue to investigate carotid plaque morphology, with its high spatial and temporal resolution.^7^

Characterization of the carotid plaque by CT has been limited in discriminating the lipid rich necrotic core from the overlying cap (made up of fibrous tissue) due to overlap of their X-ray attenuation values.^8,9^ In addition, defining plaque components based on fixed attenuation thresholds does not account for the impact of contrast timing and intensity on different tissue types that may vary between scans.^10^

The aim of this study was to determine if CT assessment of the *in vivo* carotid plaque using a novel ratio of plaque to contrast attenuation could define carotid plaque components compared to the histological assessment of the *ex-vivo* plaque following carotid endarterectomy. We then compared the ability of plaque maps created from these CT-defined components to identify histologically proven vulnerable plaques with that of previously described high-risk CT features including low attenuation plaque^7^ and ulceration.^11^

## Methods

### Data collection

In total, 70 patients from the Stroke Clinical Trials Unit at University College London that underwent both carotid CT angiography and carotid endarterectomy were included. Carotid CT angiography using a Somatom Definition 64-slice dual-source CT (Siemens, Germany) was performed in all patients prior to carotid endarterectomy. The technical parameters were Pitch, 0.20–0.48; Collimation, 32 × 2× 0.6 mm; Tube Voltage, 120 kV, and current, 360 mA and intravenous contrast (Omnipaque 350 (GE Healthcare, US) at 5 ml s^−1^. Patients underwent surgery following written informed consent after discussion in a joint neurovascular multidisciplinary team meeting. Data for this cohort were collected as part of an ongoing study that has been described in detail elsewhere.^12^ Initially, to define the X-ray attenuation values of carotid plaque components we created a derivation cohort consisting of 20 patients. Following this, the ability of CT to identify high-risk carotid plaque was examined in a validation cohort of 50 additional consecutive patients.

### Defining the X-ray attenuation of carotid plaque components

**Figure 1.**
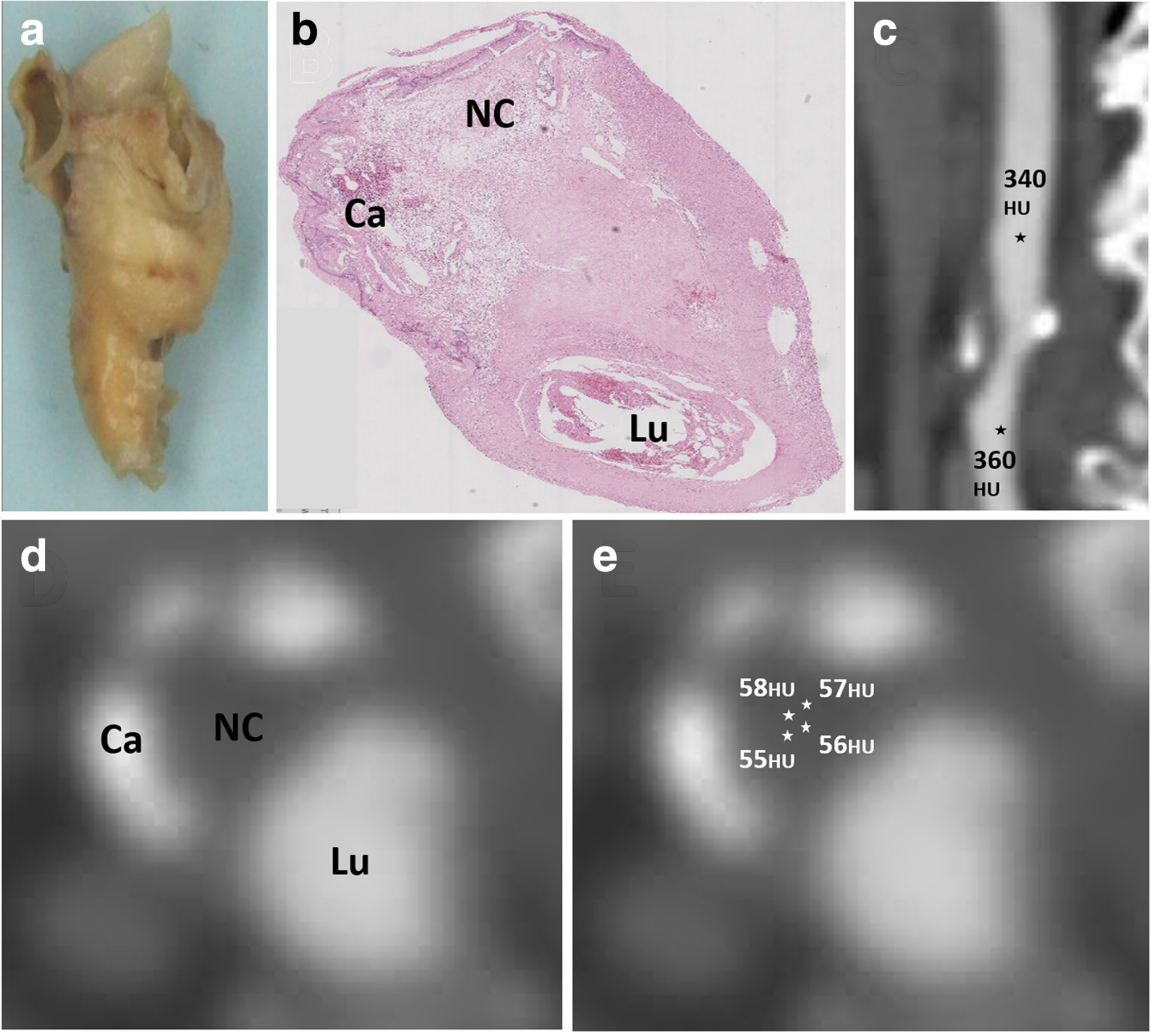
Co-registration of computed tomography (CT) with histology and attenuation sampling. (**a**) Eversion carotid endarterectomy sample removed “en-bloc”. (**b**) Plaque components pre-classified using histology (Ca – calcified plaque, Lu – lumen and NC – necrotic core). (**c**) Attenuation sampling of luminal contrast distal and proximal to plaque. (**d**) Co-registered CT cross-sectional image. (**e**) Attenuation sampling from regions of interest in necrotic core.

The 20 patients in the derivation cohort underwent eversion carotid endarterectomy. The plaque was excised intact, fixed with formalin and segmented into 3 mm blocks. Each block was embedded in paraffin and sectioned at 3 µm intervals. Plaque composition was determined using Elastin Van Gieson and Haematoxylin & Eosin stains and digital histology images were obtained by an experienced histopathologist (MJ-R). All CT analysis was performed with Vitrea^®^ Cardiovascular Software, (Vital Images Inc, United States of America). Cross-sectional carotid CT angiography images were matched with corresponding histology slides using fiduciary points such as calcified nodules (Figure 1). Plaque components were identified on the CT images utilizing the co-registered histology slides and classified as lipid-rich necrotic core, fibrous plaque and calcified plaque. Multiple regions of interests were sampled in these areas resulting in CT attenuation values for each plaque component (expressed in Hounsfield units, HU) (Figure 1). The mean attenuation of luminal contrast in each carotid was calculated using the mean of luminal attenuation measured immediately proximal and distal to each plaque (Figure 1). The ratio of plaque attenuation to its corresponding contrast attenuation was calculated for all sampled areas and used to assign ranges of plaque/contrast attenuation ratios to each plaque component.

### Computed Tomography identification of High-Risk carotid plaques

**Figure 2.**
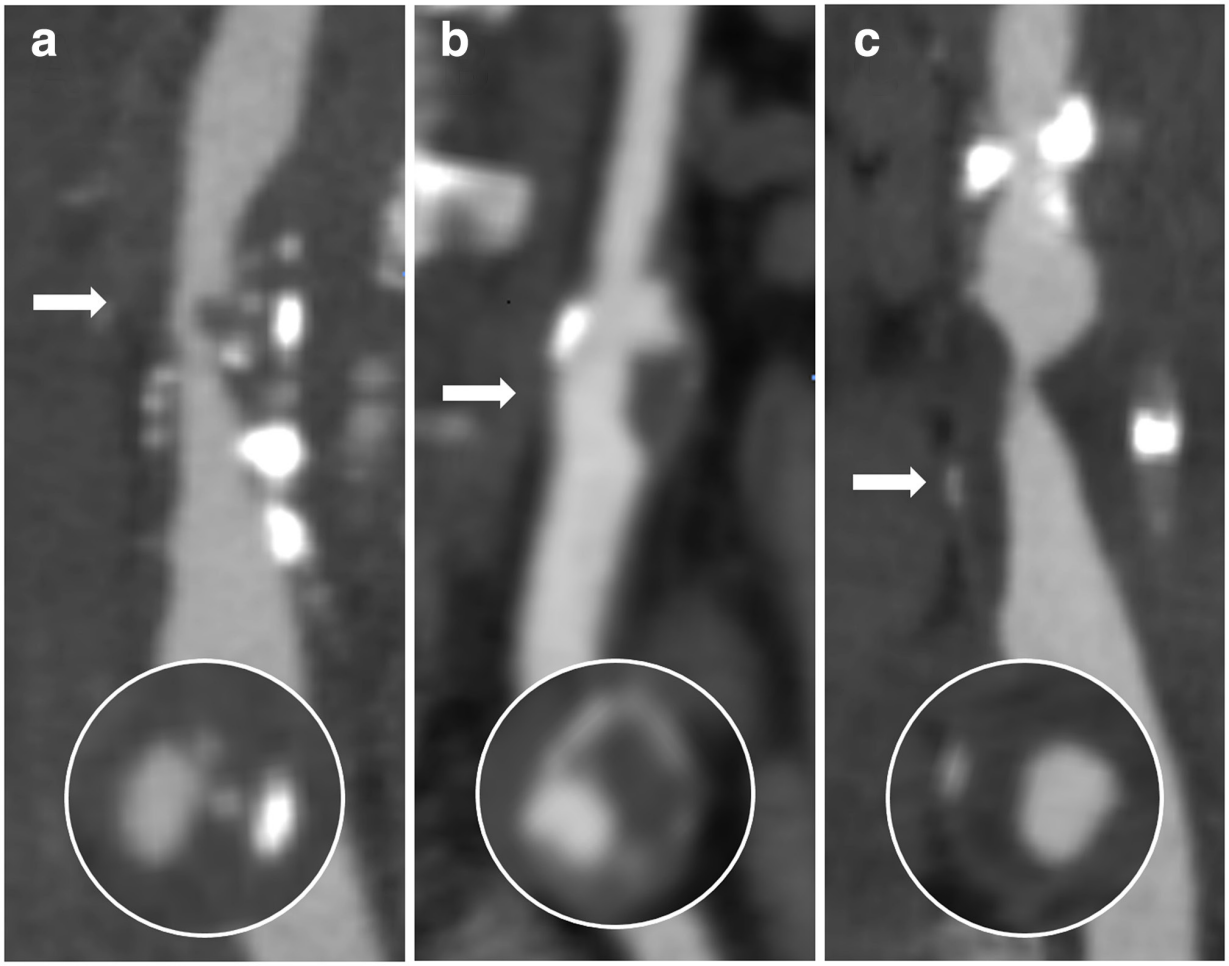
Curved multiplanar reforms of carotid artery plaque with cross-section level of arrow (inserts) of: (**a**) Ulcerated plaque, (**b**) Napkin-ring plaque and (**c**) Low attenuation plaque.

The validation cohort consisted of 50 patients who had undergone carotid endartectomy following CT carotid angiography. Curved multiplanar reformats were created of the carotid arteries to examine the plaque. CT plaque characteristics were recorded including plaque length and stenosis (calculated using the NASCET criteria.^13^ In addition, plaque burden (vessel volume – lumen volume) / lumen volume x 100% was calculated. A number of potential high-risk features have been described in carotid plaques including low attenuation plaque (LAP), ulceration and positive remodeling.^7^ This overlaps with high-risk features described in coronary plaques which also includes the Napkin-ring sign and spotty calcification.^14^ The high-risk plaque features we included in this study were defined as: LAP (area of plaque with attenuation <60 HU) corresponding to lipid-rich necrotic core,^7^ Napkin-ring sign - low attenuation plaque surrounded by high attenuation rim <130 HU^15^ and ulceration - contrast extending beyond the vascular lumen (within the plaque limits) for at least 1 mm in at least two planes^11^ (Figure 2). We elected not to include spotty calcium as there have been conflicting results on the value of plaque calcification in predicting stroke risk^16^ and we did not include positive remodeling as it may be difficult to directly compare carotid plaque from different locations as the extent of positive remodeling is segment specific.^17^

**Figure 3.**
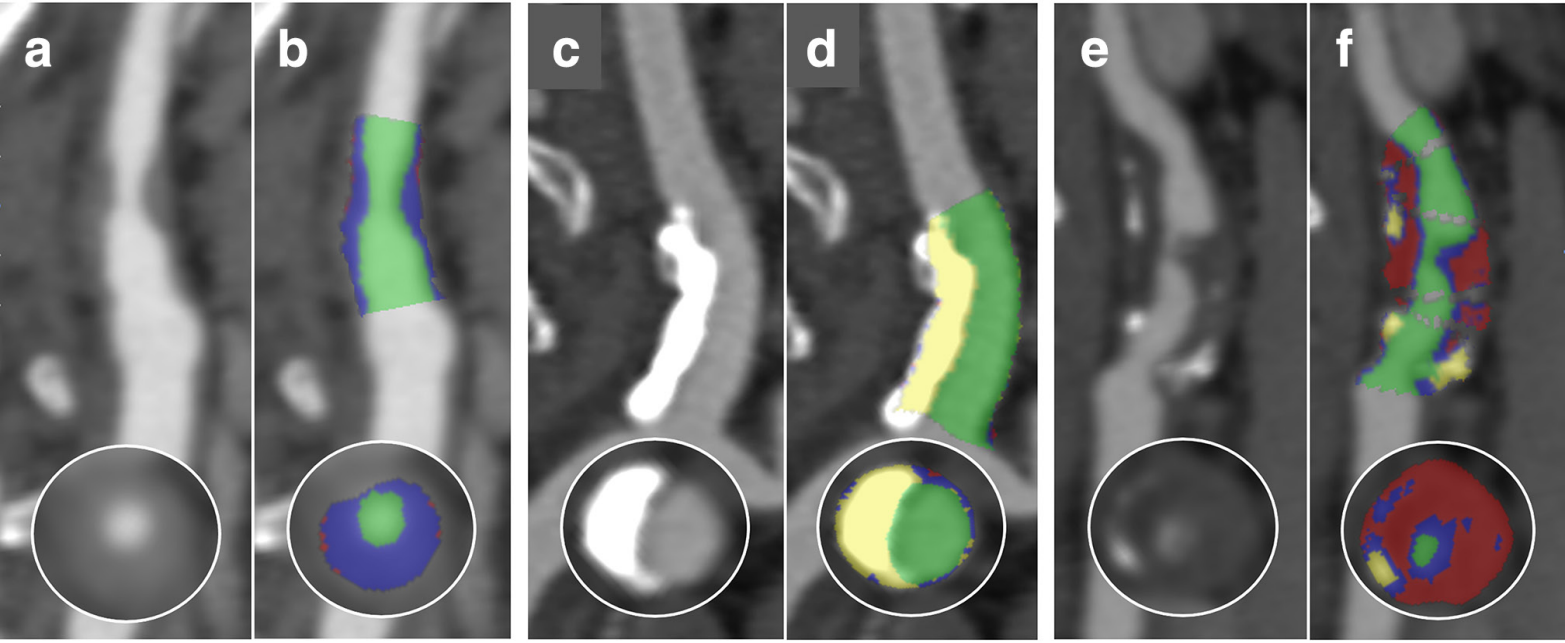
Conventional multiplanar reformat and CT Plaque Map analysis of (**a**) and (**b**) predominantly fibrous plaque, (**c**) and (**d**) calcified plaque and (**E**) and (**F**) necrotic core. With fibrous plaque colored blue, calcified plaque white, necrotic core red and lumen green.

Quantification of plaque components was performed using semi-automated Vitrea^®^ Cardiovascular Software, (Vital Images Inc, United States of America) with manual correction of vessel contours if required. Plaque was separated into constituent parts with the software colour coding each plaque voxel depending on its attenuation to create a Plaque Map (Figure 3). The attenuation (HU) cut-offs for each component were calculated according to ratios of luminal contrast and plaque attenuation determined in the derivation cohort allowing the volumes of necrotic core, fibrous plaque and calcified plaque to be calculated as well as a vulnerability index of necrotic core/fibrous plaque ratio.

Conventional and CT plaque map derived plaque characteristics were then used to compare symptomatic *vs* contralateral asymptomatic carotid plaques. Histological analysis of the carotid plaque specimens was performed following carotid endarterectomy with the plaques classified according to the American Heart Association histological classification of atherosclerotic plaques.^18^ This included type IV - Atheroma with a confluent extracellular lipid core, infiltrated with foam cells and smooth muscle cells. Type Va – Fibroatheroma (Type IV with a fibrous cap), Vb - Calcified plaque; lesion with lipid core or fibrotic tissue with large calcifications and Vc - Fibrotic plaque: fibrous connective tissue, no lipid core. The most vulnerable plaque are classified as VI - complicated plaque with possible surface defect, hemorrhage or thrombus. Conventional and CT plaque map features of histologically defined type VI plaques were compared with Type IV/V plaques.

### Power calculation

To perform a power calculation to detect histologically defined vulnerable carotid plaque using CT we used our previous work with coronary arteries which revealed CT-defined necrotic core percentages of 42.4% (SD 11.9%) in the most vulnerable (thin-cap fibroatheroma) plaque compared with 32.4% (SD 9.9%) in fibroatheroma without thin caps.^19^ Assuming similar differences in plaque component percentages in carotid plaques would require 44 plaques to detect a significant difference in the most vulnerable plaques with a power of 80% (α set at 0.05). To account for non-evaluable plaques we enrolled 50 patients in the validation cohort.

### Statistical analysis

Statistical analysis was performed using GraphPad Prism version 9 (GraphPad Software). As not all of the continuous variables were normally distributed, continuous data is presented as medians with interquartile range and compared with the Mann Whitney U test or Kruskal-Wallis test. Categorical variables are presented as percentages with accuracy expressed as point estimates of sensitivity and specificity. Diagnostic accuracy was expressed as area under receiver operating characteristic curves (ROC). Correlation between measurements was expressed as the Pearson correlation coefficient (r).

## Results

### Defining plaque components with Computed Tomography - Histological Validation

**Figure 4.**
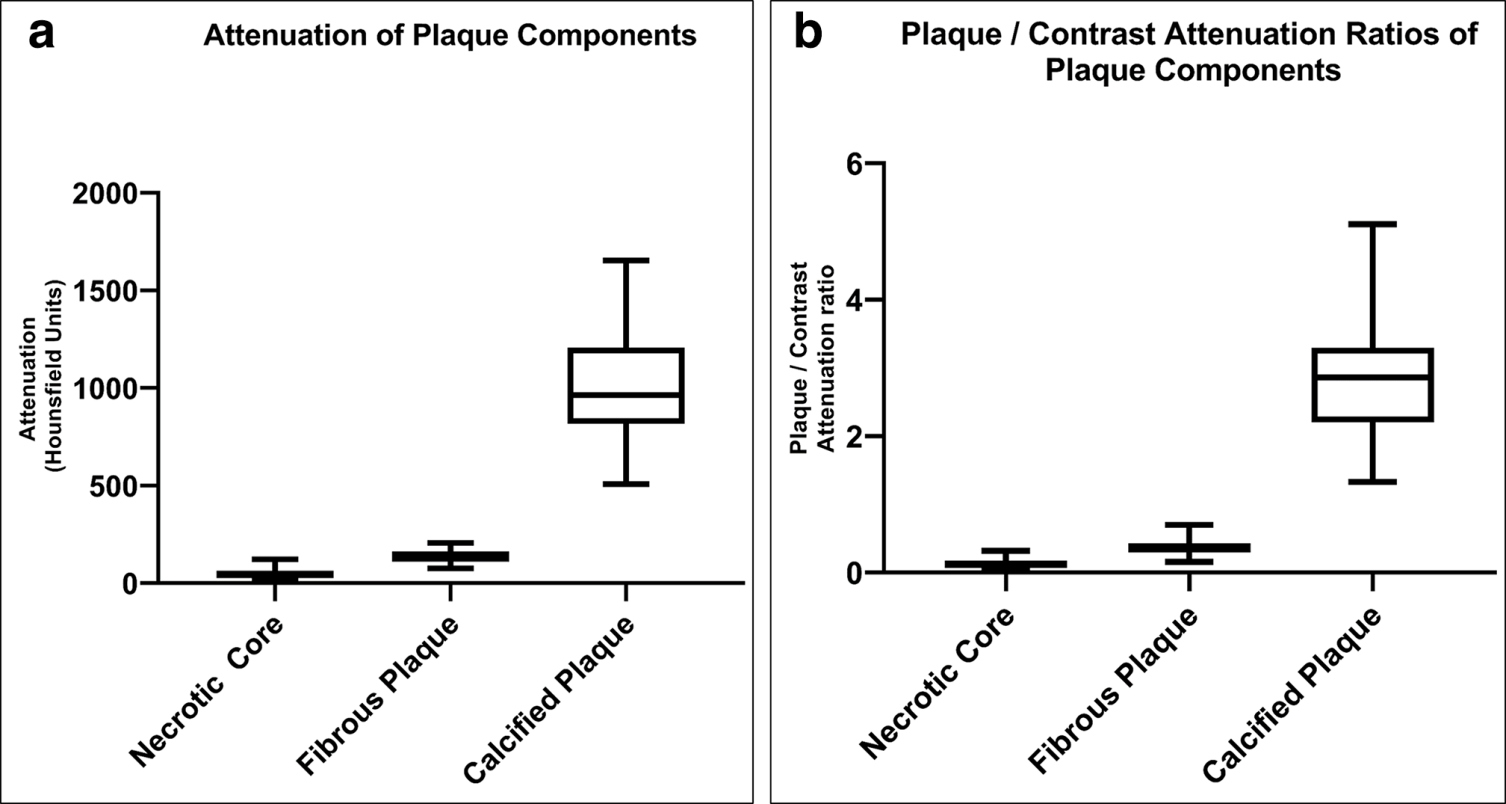
Box and whisker plot showing (**a**) attenuation values of carotid plaque components and (**b**) plaque/contrast attenuation ratios of carotid plaque components.

Of the 20 patients in the derivation cohort, co-registration of CT images and histological specimens was possible in 19 (in one patent the histological plaque specimen was too fragmented for co-registration). For these 19 patients the median age was 71(60-83) years, 42% were female and the right carotid was sampled in 13 patients and the left in 6. Attenuation sampling was obtained from 250 regions of interest in 25 plaque areas co-registered with histology. This yielded 90 attenuation values from nine necrotic cores, 70 attenuation values from seven fibrous plaques and 90 attenuation values from nine calcified plaques. The median attenuation (HU) and interquartile ranges of necrotic core 43(26-63), fibrous plaque 127(110-162) and calcified plaque 964 (816–1207) were significantly different (*p* < 0.0001) (Figure 4A). The 250 attenuation values were transformed into 250 contrast/attenuation ratios by comparing the attenuation value of the co-registered plaque components with the attenuation value of the contrast in the adjacent lumen. This gave a median contrast/attenuation ratio and interquartile ranges of 0.12 (0.07–0.18) for necrotic core, 0.36 (0.30–0.43) for fibrous plaque and 2.86 (2.20–3.30) for calcified plaque which were significantly different (*p* < 0.0001) (Figure 4B).

We then sought to determine the thresholds for plaque / contrast ratios that could be used to set attenuation thresholds providing the least overlap between plaque components. Threshold for fibrous plaque of 0.53 and calcified plaque of 1.54 allowed separation of 5–95% of values with no overlap. There was minimal overlap of the highest (95% percentile) of necrotic core values (0.23) and lowest (5% percentile) of fibrous plaque values (0.19). A threshold of 0.21 was chosen which still enabled separation of the lowest 90% of necrotic core values (10%–90% percentile) (0.05–0.21) and highest 90% of fibrous plaque (10%–90% percentile) (0.22–0.50) (Table 1).

**Table 1.** Median attenuation values with interquartile ranges and median plaque / contrast attenuation ratios used to derive attenuation ratio thresholds for carotid plaque components

Plaque Component	Attenuation (HU)	Plaque / Contrast Attenuation Ratio	5–95% Percentile	Attenuation Ratio Threshold
Necrotic Core	43(26-63)	0.12 (0.07–0.18)	0.05–0.23	0.21
Fibrous Plaque	127(110-162)	0.36 (0.30–0.43)	0.19–0.53	0.53
Calcified Plaque	964 (816–1207)	2.86 (2.20–3.30)	1.54–4.22	1.54

### Computed Tomography features of High-Risk carotid plaques

In the validation cohort, combined CT analysis and *ex vivo* histology was possible in the explanted plaque of 47/50 patients. In three patients, CT images of the carotids were not available. Of the 47 patients, 35 (74%) were male, median age was 73(66-83) years and the explanted plaque was in the right carotid in 21/47 (45%) and left in 26/47 (55%). In 44 patients, the carotid CT angiography and subsequent carotid endarterectomy were performed following a stroke or transient ischemic attack providing 44 co-registered symptomatic plaques for analysis. In four patients carotid endarterectomy was performed following an incidental finding of carotid stenosis. In addition, for the contralateral (non-operated) carotid arteries of the 47 patients eight contained no plaque and in three there was complete occlusion (no contrast distal to the plaque) preventing plaque analysis leaving 40 asymptomatic carotid plaques in total for CT analysis.

**Figure 5.**
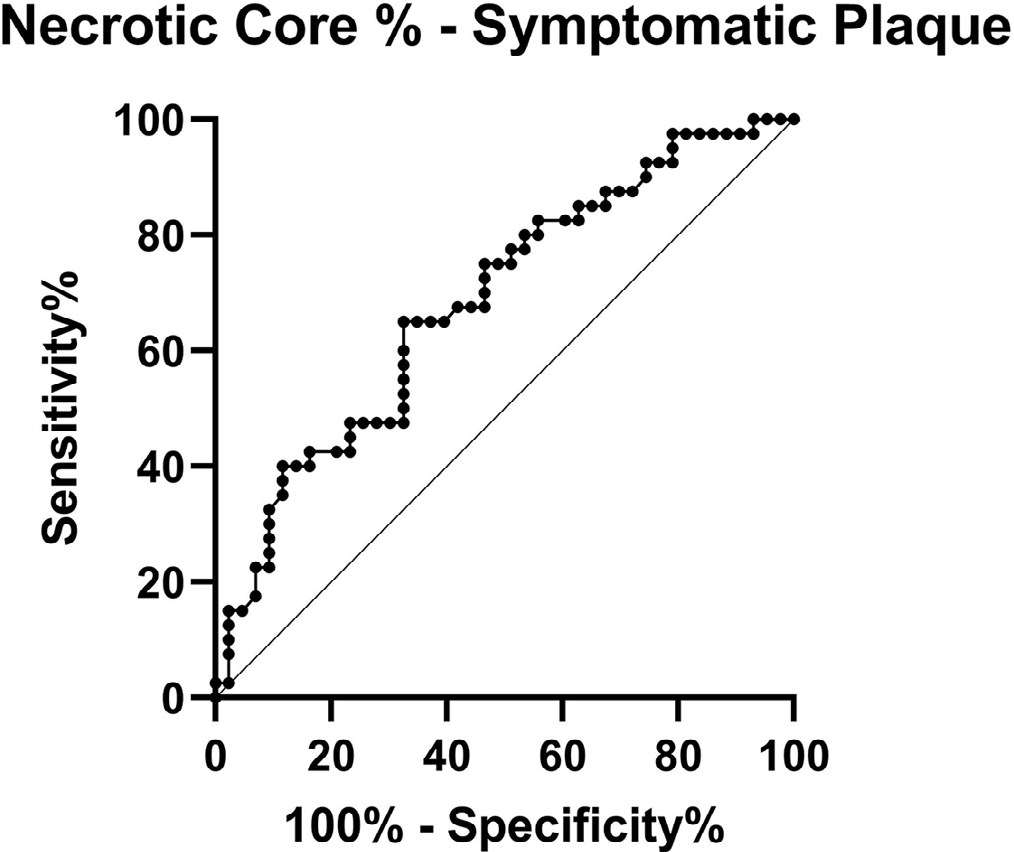
Receiver operating characteristic curve for necrotic core percentage in symptomatic carotid plaque.

Compared with asymptomatic plaques, symptomatic plaques were longer 17.9 mm(14.4–24.4) *vs* 14.4 mm(10.1–20.3), with worse stenosis 78.5%(48-90) *vs* 14.5%±(0–46.5) and greater plaque burden 62.9%(51.4–75.7) *vs* 45.2%(33.0–58.2). Conventional CT markers of vulnerability were more frequent in the symptomatic plaques (LAP 76.2% *vs* 17.5%, Napkin-ring sign 39.5% *vs* 10.0% and ulceration 31.0% *vs* 12.5%). CT Plaque Map analysis revealed that symptomatic plaques had greater percentage of necrotic core 13.5%(5.9–33.3) *vs* 7.4%(2.3–14.3) than asymptomatic plaques (Table 2). Increasing necrotic core % of the plaque was a strong predictor of being a symptomatic plaque with the area under the receiver operating characteristic curve (AUC) of 0.68, *p* = 0.004 (Figure 5). Symptomatic plaque had a higher necrotic core/fibrous plaque ratio than asymptomatic plaque (0.31 (0.17–0.70) *vs* 0.12 (0.04–0.28)). Using a necrotic core/fibrous plaque cut off of 0.5 detected symptomatic plaque with a sensitivity of 88.9% (74.7–96.6) and a specificity of 31.9% (20.4–46.2).

**Table 2.** Conventional CT analysis and CT Plaque Map analysis of symptomatic and asymptomatic carotid plaques (LAP – low attenuation plaque, NC – necrotic core, Fi – fibrous plaque, Ca – calcified plaque, NC/Fi – necrotic core / fibrous plaque ratio)

	Plaque Type		
	Symptomatic (43)	Asymptomatic (40)	*P* value
Conventional CT			
Lesion length (mm)	17.9 (14.4–24.4)	14.4 (10.1–20.3)	0.002
Lesion stenosis	78.5%(48-90)	14.5%±(0–46.5)	<0.0001
LAP	76%	18%	<0.0001
Napkin Ring	40%	10%	<0.0001
Ulceration	31%	13%	0.04
Plaque quantification			
Vessel volume (mm^3^)	905.5 (694.6–1335)	674.9 (466.6–1119)	0.11
Plaque burden	62.9%(51.4–75.7)	45.2%(33.0–58.2)	<0.0001
CT Plaque Map			
NC volume (mm^3^)	94.3 (22.4–251.3)	20.7 (8.0–58.8)	<0.0001
NC %	13.5%(5.9–33.3)	7.4%(2.3–14.3)	0.004
Fi volume (mm^3^)	249.0 (159.1–335.3)	150.9 (102.9–214.7)	<0.0001
Fi %	43.4%(27.7–53.4)	51.3%(31.9–66.2)	0.20
Ca volume (mm^3^)	214.0 (34.8–427.0)	101.2 (19.4–263.5)	0.10
Ca %	25.7%(7.6–66.4)	38.5%(15.3–58.7)	0.57
NC/Fi	0.31 (0.17–0.70)	0.12 (0.04–0.28)	0.0001

### CT analysis of histologically defined vulnerable plaque

Of the 47 carotid plaques removed by carotid endarterectomy and undergoing histological analysis, 24 were classified as the most vulnerable (Type VI). Of the remainder 23 were Type V (6 - Va, 13 - Vb, 4 – Vc) and one was Type IV). The patients with the most vulnerable (Type VI) were similar in age and gender to those with other plaques. Conventional CT parameters were no different between Type VI plaque and other plaque in lesion length, stenosis or plaque burden and there was a similar rate of low attenuation plaque and Napkin-ring sign (Table 3). There was no significant correlation between plaque stenosis and necrotic core % in these symptomatic plaques (*r* = 0.16, *p* = 0.29). Compared to the other plaque types, Plaque map analysis revealed that Type VI plaque had a greater volumes of necrotic core (148.5 mm^3^(54.5–380.6) *vs* 44.1 mm^3^(19.6–139.0) and fibrous plaque (274.8 mm^3^(175.6–457.5) *vs* 194.6 mm^3^(136.7–309.5). There was no difference in volume of calcified plaque between the plaque types. Type VI plaque had numerically but not statistically significant greater percentages of necrotic core than the other plaque types (20.4%(9.2–42.7) *vs* 9.8%(5.0–26.3)) and using a necrotic core/fibrous plaque cut off of 0.5 detected Type VI plaques with a sensitivity of 75.0% (55.1–88.0) and a specificity of 39.1% (22.2–59.2).

**Table 3.** Conventional CT analysis and CT Plaque Map analysis of histologically defined Type VI and Type IV/V carotid plaques (LAP – low attenuation plaque, NC – necrotic core, Fi – fibrous plaque, Ca – calcified plaque, NC/Fi – necrotic core / fibrous plaque ratio)

	Plaque Type		
	Type IV/V^20^	Type VI^21^	*P* value
Age	75(67-83)	71(64-83)	0.35
Sex (male)	64%	79%	0.10
Conventional CT			
Lesion length (mm)	17.2 (13.9–23.4)	18.2 (15.2–26.5)	0.25
Lesion stenosis	67%(28-90)	77%(52-90)	0.60
LAP	71%	82%	0.71
Napkin Ring	38%	48%	0.28
Ulceration	13%	57%	0.0002
Plaque quantification			
Vessel volume (mm^3^)	864.8 (621.1–1154)	1013(721.9–1335)	0.51
Plaque burden	61.0%(50.7–75.1)	69.5%(54.2–75.0)	0.43
CT Plaque Map			
NC volume (mm^3^)	44.1.(19.6–139.0)	148.5 (54.5–380.6)	0.02
NC %	9.8 (5.0–26.3)	20.4%(9.2–42.7)	0.09
Fi volume (mm^3^)	194.6 (136.7–309.5)	274.8 (175.6–457.5)	0.04
Fi %	42.8%(24.3–59.4)	43.9%(31.0–52.5)	0.65
Ca volume (mm^3^)	175.5 (25.4–453.6)	214.0 (35.3–424.9)	0.87
Ca %	28.3%(7.7–68.3)	22.9%(4.6–60.8)	0.52
NC/Fi	0.24 (0.13–0.58)	0.4 (0.27–0.93)	0.07

CT identified 13/23 histologically defined ulcerated plaques correctly and excluded 18/24 correctly giving a CT a sensitivity to detect ulcerated carotid plaques of 56.5% (34.5–76.8) and specificity 87.5%(67.6–97.3).

## Discussion

### CT attenuation of plaque components

In this study, we defined the CT attenuation of plaque components using co-registered histological samples. All carotid endarterectomies in the derivation cohort were performed by eversion enabling minimal plaque disruption facilitating the co-registration with CT. We were able to identify attenuation ranges for the different plaque components that had minimal overlap. We identified the mean attenuation of necrotic core as 50 HU, similar to that found in previous studies of 40 HU^8^ and 60 HU.^9^ Importantly, the attenuation of plaque is altered by contrast intensity,^10^ with fatty plaque potentially the most affected by contrast enhancement.^22^ To account for this we defined necrotic core according to the ratio of plaque to contrast attenuation. Defining plaque components based on the ratio of plaque attenuation to contrast attenuation automatically adjusts for interpatient variations in contrast intensity, a technique that has also been used in coronary arteries identifying plaque components with good diagnostic accuracy.^23^ The attenuation ratios of necrotic core and fibrous plaque had minimal overlap – important as the necrotic core/fibrous plaque ratio has previously been shown to correspond with vulnerable coronary plaque.^19^

### CT analysis of symptomatic carotid plaque

Compared with asymptomatic plaques, symptomatic plaques were longer, had greater plaque burden and were more stenotic. However, stenosis alone is an unreliable of predictor of future events with the majority of ischemic events occuring in patients with moderate stenosis^24^ so identification of plaque features that may predict vulnerability to disruption is important to guide clinical decision making.^7^ Interestingly, whilst we found that symptomatic plaques had a greater percentage of necrotic core than asymptomatic, there was no correlation between stenosis and necrotic core %. Imaging features of carotid plaque vulnerability previously linked with future stroke include lipid-rich necrotic core, plaque volume, neovascularization and inflammation, ulceration and intraplaque hemorrhage.^21^ We found symptomatic plaques were significantly more likely to contain low attenuation plaque, the napkin ring sign and ulceration. Low attenuation plaque (using the accepted attenuation cut-off of 60HU) is associated with lipid-rich necrotic core, a feature of vulnerable plaque.^9^ The Napkin-ring sign (low attenuation plaque surrounded by a rim of higher attenuation plaque has not previously been investigated in the carotid, however it is strongly linked to vulnerable coronary plaque.^15^ The cause of the higher attenuating plaque is not clear but carotid plaque enhancement has been shown to be related to neovascularization, another feature of vulnerable plaques.^20^ Finally, carotid plaque ulceration is known to be a predictor of future ischaemic stroke.^7^ This study demonstrated detection of ulceration with sensitivity 57% and specificity 88%. This is lower than previously described in a previous series – sensitivity 94%, specificity 99% however this was a comparison of macroscopically identified rupture^25^ as opposed to ours which was validated against microscopic histology and hence smaller ulcers will be identified which may be missed by CT due to its spatial resolution.

### CT plaque maps of vulnerable plaque

The most advanced atherosclerotic plaques defined by the American Heart Association as type VI are characterized by intraplaque haemorrhage, thrombus or a ruptured fibrous plaque.^26^ These plaques when identified by MRI are the most associated with stroke.^27^ We investigated the ability of CT to distinguish histologically proven type VI plaques.

Using conventional CT parameters we found no difference in lesion length, overall plaque burden, presence of low attenuation plaque or the Napkin-ring sign but did detect ulceration in type VI plaques more frequently than non-type VI (57% *vs* 13%). This is understandable as ulceration is one of the features of type VI plaques. Another feature is intraplaque hemorrhage, which is strongly associated with stroke.^28^ Unfortunately, at present CT detection of intraplaque hemorrhage is limited by its attenuation overlap with other plaque components.^29^ Recently the presence of adventitial calcium – the “thin rim sign” on carotid CT angiography has been shown to be associated with intraplaque hemorrhage and may be a useful marker of vulnerability.^30^ A cardinal feature of plaque prone to rupture identified post mortem is the presence of a thin fibrous cap.^31^ Due to limitations of spatial resolution, CT is unable visualize directly the thin fibrous cap, although a high plaque map ratio of necrotic core / fibrous plaque is a marker for thin-capped fibroatheroma in coronary arteries.^23^ Plaque map analysis of necrotic core in the carotid plaques that underwent histological classification revealed that despite similar overall plaque volume, type VI plaques had larger necrotic cores.

## Limitations

We defined the attenuation of plaque components based on the gold standard of *ex-vivo* histology. However, despite utilizing eversion endarterectomy to keep fragmentation of histology samples to a minimum it is possible that co-registration may have been affected by changes in the plaque that occurred during excision and processing. In addition, whilst using ratios of contrast to plaque attenuation rather than fixed attenuation ratios should adjust for interscan variation in contrast intensity, caution should be applied when extrapolating these to other contrast protocols where different flow rates are used. It should also be noted that this is a single-centre study with all scans performed on a single CT scanner. Further validation at multiple institutions using different CT scanner vendors is required for more widespread utilization. Finally, contralateral plaques of patients undergoing carotid endarterectomy have been assumed asymptomatic. However, it is possible that they will have been responsible for previous or clinically silent events.

## Conclusion

Carotid plaque components can be differentiated by CT using a ratio of plaque/contrast attenuation. The CT-derived plaque map volumes of necrotic core helped distinguish the most advanced (Type VI) plaques and the ratio of necrotic core to fibrous plaque may provide a new marker of plaque vulnerability.
